# Assessment of Nutrient and Food Group Intakes across Sex, Physical Activity, and Body Mass Index in an Urban Brazilian Population

**DOI:** 10.3390/nu10111714

**Published:** 2018-11-09

**Authors:** Alessandra Gaspar Sousa, Teresa Helena Macedo da Costa

**Affiliations:** 1 Human Nutrition Graduate Course, School of Health Science, University of Brasilia, 70919-970 Brasilia, Brazil; 2 Department of Nutrition, School of Health Science, University of Brasilia, Darcy Ribeiro Campus, 70919-970 Brasilia, Brazil; thmdacosta@gmail.com

**Keywords:** physical activity, body mass index, adults, dietary assessment

## Abstract

The burden of preventable diet-related diseases is significant and becoming worse. Thus, accurately assessing food intake is crucial to guide public health policies and actions. Using food dietary recalls, we evaluated usual dietary intake according to physical activity and nutritional status in an adult urban population from Brasília, Brazil. The usual nutrient and energy intakes distributions were estimated using the Iowa State University (ISU) method. Energy and nutrient intakes were stratified by gender, age group, body mass index (BMI), and physical activity (PA). The prevalence of inadequate intake was highest for vitamins E and D. Both men and women had excessive sodium intake. The percentage of intakes below daily serving recommendations for food groups were 96% for cereals, 74% for vegetables, and 87% for dairy products, whereas percentage of intakes above daily serving recommendations were 97% for meat, 93% for pulses, and 99% for fat/oils. Energy and nutrient intakes were highest in overweight and physically active individuals within the categories of BMI and physical activity, respectively. Our study found that high-income urban Brazilians consume large quantities of meat, beans, fat/oils, and exhibit a low prevalence of nutrient inadequacies but have excessive sodium intake. Energy and nutrient intakes are highest among men, as well as overweight and physically active individuals.

## 1. Introduction

The increasing globalization and urbanization have influenced dietary patterns and the lifestyles among different populations worldwide. Modern lifestyles have led to dietary changes characterized by insufficient fiber intake and increased consumption of processed and ultra-processed foods with high energy density, which are associated with increased risk for development of non-communicable diseases (NCDs) [1,2]. In addition, social determinants, such as education, occupation, income, gender, and ethnicity, are strongly correlated with the prevalence of NCDs and risk factors, such as tobacco use, alcohol abuse, and physical inactivity [3].

A healthy diet with a wide variety of foods helps provide a range of different nutrients to the body. Micronutrients play an important role in preventing diseases and have a significant impact in Brazil. The action of some vitamins can help reduce the occurrence of NCDs [4]. Vitamin D and calcium are essential for muscle skeletal health and reduce the risk of osteoporosis [5]. However, excessive sodium intake is associated with high blood pressure and increased risk of cardiovascular and renal disease [6].

The adequate assessment of food intake practices is critical to determine dietary trends and assess the effects of interventions at the population level. One goal of measuring dietary intake is to capture usual or habitual intake or a person’s dietary consumption over extended periods of time [7]. Different analytic methods for estimating usual intake have been developed to address the issue of within-person variation found in dietary intake data as assessed by 24-h recalls [8]. Nevertheless, measuring dietary intake is challenging because food consumption is a complex phenomenon which makes food intake data notoriously difficult to analyze.

Because trends in food intake can predict the nutritional and health status of the population and serve as a baseline to guide public health policies and actions, this study aimed to evaluate usual dietary intake according to individuals’ physical activity and nutritional status in an urban population.

## 2. Materials and Methods

### 2.1. Sample Design

This study is a cross-sectional population-based survey of the adult population of Brasília, Federal District, Brazil. The Federal District is one of 27 federal units of Brazil, consisting of the country’s capital (Brasília) and several other satellite cities and communities covering an area of 5801.9 km² and with an estimated population of 2.6 million people in 2010 [9].

The sample size was based on the number of households registered at the Energy Company of Brasília (CEB), which serves 100% of the households in the city. We used a stratified cluster sampling design, considering the household as the primary sampling unit with the probability of selection proportional to the size of the four sanitary regions of central Brasília. We followed the sample protocol used in a previous study on physical activity and dietary knowledge from our research group [10]. A random sample of households was selected from each region. Residents were invited to participate with a letter mailed to their residence, then a study interviewer visited each selected household. A replacement sample for vacant households and those that refused to participate was drawn from the CEB address list and neighboring households within the four city regions. Sample size was based on an alpha error of 5% and assuming that 80% of the population engaged in less than 150 min of physical activity per week [10]. The total number of households recruited in our study was 429 distributed across the four city regions: South Wing (185 households, 43%), North Wing (211 households, 49%), Planalto Village (13 households, 3%), and Urban Military Sector (20 households, 5%).

The study was conducted from February 2016 to July 2017. The number of individuals interviewed was 506, of whom 363 (72%) were recruited from the replacement sample. Chi-square test of age (*p* = 0.95) and Body Mass Index (BMI) (*p* = 0.39) categories showed no significant differences between the random and replacement samples, which indicates that both samples belonged to the same population.

Inclusion criteria were residing in the selected household, age 20 years or older, and agreeing to participate in the study. Non-residents of the selected household, pregnant women, nursing mothers, individuals with special needs, and disabled or mentally handicapped persons who were unable to describe their dietary habits or who could not be measured on a scale and anthropometer were excluded from the study. All procedures were approved by the Research Ethics Committee of the University of Brasília School of Health Sciences (Protocol n.1.350.858 CAAE 48418315.4.0000.0030). The participants were properly informed of the purpose of the study and provided written informed consent.

### 2.2. Sociodemographic Variables and Anthropometric Data

Sociodemographic characteristics included age, gender, socioeconomic status, years of schooling, and physical activity. The socioeconomic status was assessed using the Brazilian Economic Classification Criterion on the sociodemographic data questionnaire [11]. The total time of physical activity and the types of physical activity performed over two nonconsecutive 24-h periods were recorded on a form. To be considered physically active, the individual must do 150 min per week of physical activity. BMI was calculated from measured weight and height, and the World Health Organization (WHO) BMI cut-offs were used to classify the respondents. Because of the small number of individuals (1.6%) in the low-weight BMI group, it was combined with the eutrophic group. The obese category was not divided into subgroups. This resulted in three BMI categories: <25 kg/m² = eutrophic; 25‒29.9 kg/m² = overweight; and ≥30 kg/m² = obese [12]. As the +70-year-old age group (4.2%) was also too small and based on Estimated Average Requirement (EAR) cut-off points, the 50‒70-year-old age group was combined with the oldest group. These combined classifications helped to increase the sample size in each subgroup and perform the usual intake analysis.

### 2.3. Dietary Intake

Food intake was estimated from two nonconsecutive 24-h food recalls, in which individuals reported all food and beverages consumed in the previous 24 h. Meal times, recipe preparation methods, and serving sizes were carefully documented. To assist the respondent describe the amount of food and beverages consumed and the estimating portion size, we used real tableware objects (cups, glasses, and cutlery), and a food photography booklet [13]. The first food recall was conducted in person and the second was conducted by telephone. The Nutrition Data System for Research (NDSR) software (Nutrition Coordinating Center, University of Minnesota, Minneapolis, MN, USA) was used to estimate dietary nutrient content from food recall data. The NDSR is based on the United States Department of Agriculture (USDA) data, thus the Brazilian foods that were not included in the program database had their nutritional value inserted or adapted from a food of the same value according to national information [14].

The energy provided by each food item were obtained from NDSR output and grouped according to the food groups determined by the 2006 Brazilian National Dietary Guidelines [15]. The newer version of the Brazilian Dietary Guideline published in 2014 was not used because it classified foods according to the degree of processing of food items [16]. The distribution of energy considering a 2000 kcal diet and the number of recommended daily servings from each serving group included: (a) bread, cereals, and tubers, 120 kcal/serving, recommended six servings/day; (b) vegetables, 15 kcal/serving, recommended three servings/day; (c) fruits, 35 kcal/serving, recommended three servings/day; (d) meats, 190 kcal/serving, recommended one serving/day; (e) dairy products, 120 kcal/serving, recommended three servings/day; (f) beans, lentils, and nuts, 55 kcal/serving, recommended one servings/day; (g) oil and fats, 73 kcal/serving, recommended one serving/day; and (h) sugars (total sugar) and candies, 110 kcal/serving, recommended one serving/day.

### 2.4. Statistical Analysis

Dietary intake analysis was performed by implementing the Iowa State University (ISU) method using the PC Software for Intake Distribution Estimation (PC-SIDE version 2.0, 2017; Department of Statistics, Iowa State University, Ames, IA, USA) to estimate the distributions (mean and percentiles) of usual intakes of nutrients and energy from food [17]. In addition, nutrient intake was stratified by gender, age group, BMI, and physical activity. The ISU method was chosen because of its ability to improve estimates for usual dietary intake of energy and nutrients by considering the within-person variance of intake [16].

For nutrients with asymmetric distribution, such as copper, selenium, vitamins A, B2, and B3, we used different statistical methods to adjust the usual intake distribution in the following order of priority. (a) The within- and between-person variation and the kurtosis of the distribution of the measurement error were obtained from the estimated usual intake distribution for the nutrient without the outliers. The new values (within- and between-person variation, and kurtosis) were then applied to the PC-Side configuration file to run the full model including the outliers [18]. (b) Reducing the accuracy of the Anderson-Darling statistical test to adjust the distribution (c) The outliers were replaced by the values corresponding to the mean ± 3 SD (standard deviation). In addition, recall order (first in person or second by telephone) and sample (random or replacement) were included as covariates in all PC-SIDE analyses to control for any differences in interview order or sampling.

The prevalence of inadequate micronutrient intake was estimated according to gender and age group using the Estimated Average Requirements (EAR) as set by the Institute of Medicine, Food and Nutrition (IOM, USA) except in the case of iron [5,6,19,20,21,22]. Because the distribution of iron requirement for women of reproductive age is skewed, estimation of iron inadequate intake was calculated using the probabilistic approach [23]. For women 19‒30 and 31‒50 years old, the prevalence of inadequate iron intake corresponds to the sum of the percentage of individuals with inadequate intake in each percentile. For men and older women, the percentiles of the usual iron distribution were estimated, and a probability of inadequacy was specified for each percentile, as recommended by the IOM (2000) [22].

Mean intake was compared to adequate intake (AI) values for nutrients that an EAR has not been established, as it was not possible to estimate the prevalence of inadequate intake [6]. Values above the tolerable upper intake level (UL) were also considered [6,23].

Therefore, intakes lower than the EAR indicate the estimated prevalence of inadequate intake within a group. The proportion of the population with intake greater than the UL identifies those with excessive intake, who are potentially at risk of adverse effects, whereas intake above the AI define the prevalence of adequate intake for the group.

The R statistical program (R Development Core Team, 2016) was used to adjust the food group database. The data for vegetables and pulses for the obese group had a very asymmetric distribution, and the analyses could not be performed in the PC-SIDE software even with prior adjustments in the R program. A Chi-square test was applied to compare the proportion of the food group servings below the Brazilian National Dietary Guidelines for the BMI and PA categories.

Data organization and descriptive statistics (averages, standard deviation, histograms, and frequency distributions) were performed using Statistical Analysis System (SAS) software version 9.4 (SAS Institute Inc., Cary, NC, USA).

## 3. Results

Sociodemographic characteristics and percentage of energy provided by macronutrients are presented in Table 1. The mean age of the population was 40 years old (standard deviation [SD] = 15.6 years), and for the Dietary Reference Intake (DRI) life stage groups, the mean age was 25.0 ± 2.99, 39.0 ± 5.69, and 60.0 ± 7.8 years old in the female group; and 25.0 ± 3.09, 39.0 ± 6.0, and 62.0 ± 8.81 years old in the male group. In addition, 87% of the sampled population had completed higher education (>15 years of schooling) and 76% had high/medium socioeconomic status (monthly personal income >US$ 2,102.94 or R$ 7,799.5 Brazilian Reais) (Table 1).

### 3.1. Usual Dietary Intake

#### 3.1.1. Macronutrients

As expected, men consumed more calories from protein, carbohydrates, and lipids than women (Table 1). Macronutrient calorie intake had a balanced distribution: 47.0 ± 54.4% carbohydrates, followed by 19.4 ± 28.3% protein, and 32.2 ± 16.2% lipids. The amount of food and beverages consumed was 3847 ± 892.8 g and 3024 ± 673.2 g in men and women, respectively.

#### 3.1.2. Micronutrients

Usual nutrient intake was estimated for 22 micronutrients, in which 11 had low prevalence of inadequacy and no excessive intake (<10% below the EAR and UL): phosphorus, magnesium, copper, selenium, potassium, vitamins K, B1, B2, B3, B5, and B6. Vitamin B5 was above the AI for more than 75% of men and 25% of women. Potassium was above the AI in 5% of men only, but potassium intake below the AI cannot be evaluated (Supplementary Materials Table S1).

Nutrients with a prevalence of inadequate or excessive intake greater than 10% are shown in Table 2. Both men and women had excessive sodium intake, which was found in 98% of men 20‒50 years old and 95% of men over 50 years old. Excessive manganese intake was also found in men 20‒50 years old (26%).

The prevalence of inadequate intake, in both men and women, was highest for vitamins E and D. The highest prevalence of inadequate intake of vitamin E and D was found in women 20‒50 years old (99% of inadequacy for both nutrients). The prevalence of inadequate intake of calcium and folate was also high among women (35% and 62%, respectively). The prevalence of inadequate intake of zinc and vitamin C was similar between the genders (Table 2).

Even though women of childbearing age are vulnerable for iron deficiency, the prevalence of iron inadequacy was only 11% in women 20‒50 years old and intake in women in the fifth percentile was within the reference value.

### 3.2. Usual Dietary Intake According to BMI and Physical Activity

The largest amount of food was consumed by overweight (3556 ± 816.8 g) and physically active (3467 ± 878 g) individuals in the BMI and physical activity categories, respectively (Supplementary Materials Table S5). Overweight individuals consumed 45% of energy from carbohydrates, 20% of energy from protein, and 33% of energy from lipids. Physically active individuals consumed 46% of their energy from carbohydrates, 20% from protein, and 33% from lipids.

The distribution of usual intake of nutrients revealed that intake was greater among overweight and physically active individuals for most nutrients analyzed. The highest amounts of manganese and vitamin C were found in obese individuals, whereas eutrophic individuals had the highest intake of vitamin K. Additionally, physically inactive individuals had the highest intake of vitamin C, with 5% of the physically inactive individuals consuming over three times more vitamin C than active individuals (1899 ± 1220 mg × 576 ± 116 mg, mean ± SE; Supplementary Materials Table S3).

### 3.3. Food Groups

The usual intake distribution of food groups revealed that the prevalence of inadequate intake of cereals (below the recommended six daily servings) was greater than 90% across all categories. Conversely, intake of meat and pulses exceeded the recommended one serving across all categories (Table 3).

The prevalence of inadequate intakes of fruits, vegetables, and pulses (below the recommended daily intake of three servings) was significantly higher in physically inactive individuals. Conversely, consumption of sugars was significantly higher among eutrophic and physically active individuals. In addition, most of the sample had more than one daily serving of the fat and oil group (Table 3). 

## 4. Discussion

Our survey of nutrient and food groups consumed by the urban population living in Brasília found that both genders had a high prevalence of inadequate intakes of vitamin E and vitamin D, as well as excessive sodium intake. The role of vitamin D in skeletal muscle and bone health is well established. However, over the last decade evidence has suggested that low vitamin D levels are associated with a number of non-skeletal disorders including cancer, heart disease, high blood pressure, diabetes, age-related cognitive decline, Parkinson’s disease, multiple sclerosis, and arthritis [24]. Vitamin D is consumed in the diet via the intake of dietary supplements and animal-based foods and synthesized in the skin through the action of sunlight, specifically ultraviolet B (UVB) radiation [5]. UVB sunlight exposure, rather than diet, has been reported as the main source of active vitamin D for the majority of the population [25]. Additionally, DRI recommendations for vitamin D are based on the US population’s exposure to the sun, which is lower than in Brazil [5]. Thus, intake requirements for vitamin D in the Brazilian population may be overestimated.

The prevalence of inadequacy was very similar for both genders. Mean dietary intake of vitamin D in our study was slightly higher than in other Brazilian studies [26,27]. European intake were looked at in an EPIC (European Prospective Investigation into Cancer and Nutrition) study, and for all countries combined, the daily mean vitamin D intake was reported as 4.8 μg/day for men and 3.3 μg/day for women, but with considerable variation between countries [28]. However, accurate comparison of vitamin D intake is hampered by different study methodologies, variations in dietary assessment techniques, different age classifications, and limitations in food composition tables.

In the diet, α-tocopherol is found in foods such as nuts and seeds and in vegetable oils such as wheat germ, sunflower seed, safflower, and olive [29]. Even though our data indicate that these foods are part of the Brazilian diet, nearly the entire sample did not consume sufficient dietary vitamin E to meet the EAR. This result suggests that the recommended EAR for vitamin E is too high and that the apparent low dietary intake of α-tocopherol found in our study may be due to underreporting of vegetable-oil that is use in Brazil for cooking and is very difficult to estimate.

Inadequate intake rates of zinc, vitamin D, vitamin E, and vitamin B12 estimated in our study are comparable to those described in a nationwide survey in Brazil, whereas the prevalence of iron inadequacy presented in our study was lower [26]. Other studies of the European population found average micronutrient intakes that were lower than those observed in our study. Similar to our findings, a 2013 Spanish study showed higher intake of zinc, selenium, folate, vitamin B12, and vitamin E in men than in women in the entire population; however, mean nutrient intake was higher in our study than in the Spanish one [30,31]. A review of macro- and micronutrient intake in European adults also found median intakes of folate, vitamin D, and calcium that were lower than those observed among Brazilian adults [32]. These differences could be due to variations in Brazilian eating habits, the statistical methods used to adjust the usual intake distributions, and the different recommended daily intake between the countries.

Alternatively, extremely high manganese intake was found in our study compared to other findings in several countries [33]. Our results for manganese could be related to the high tea consumption observed in our study, especially Yerba Mate. Rusinek-Prystupa et al. (2016) found that Yerba Mate teas can supplement the diet with valuable microelements, in particular iron and manganese [34].

As expected, energy and macronutrient intakes declined progressively with age. The first nationwide survey evaluating food intake in Brazil also showed that men consumed almost 23% more calories than women, irrespective of region or location of residence [26]. In a study of the urban population from eight Latin American countries showed, men reported higher energy intake than women, independent of age group and country [35]. Similar to our findings, energy intake in all countries was highest in young men and lowest in older women. However, the estimated mean daily total energy intake in our study was lower than that of the United States (US) adult population [36]. This difference could be explained by variations in eating habits, differences in dietary assessment methodologies, or differences in age/gender distributions.

Improving the quality of the diet by including a higher proportion of cereals, legumes, fruit, vegetables, and dairy products along with reducing the amount of highly ultra-processed food may help reduce the prevalence of vitamin and mineral inadequacies. The usual intake distribution of food groups showed that more than 50% of respondents in our study consumed fewer fruits, vegetables, and dairy products than recommended, which explains the high prevalence of inadequate intakes of vitamin C, folate, and calcium as well as the low prevalence of potassium intake above the AI. Moreover, the high intake of sugars and the excessive intake of sodium (above the tolerable limit) observed in our study may be due to the increased contribution of ultra-processed foods in the Brazilian diet [37].

A study comparing national dietary data from the US and Brazil showed that dietary intake in the US had frequencies two times higher than those in Brazil for milk, dairy products, deli and cured meat, savory snacks, cereals and grains, sweets and confections, and soft drinks. However, Brazil had higher intake frequencies for meat, beans, and legumes, similar to those found in our study [38]. In addition, the Latin American Study of Nutrition and Health (ELANS) found a large contribution from refined carbohydrates, fat- and sugar-rich foods and beverages, and limited contribution from complex carbohydrates as well as fruits and vegetables [35].

In Brazil, a limited-variety diet that relied on the consumption of rice and beans has been identified as a traditional dietary pattern among Brazilian adults and a protective factor for obesity [38,39]. The emergence of tailored diet regimens for weight reduction consisting of high-fats, moderate-proteins, and very-low carbohydrates [40], especially among individuals of high socioeconomic level, may explain the high proportion of individuals (90%) in our study who consumed less than the recommended daily serving of cereals and more than the recommended servings of meat, fat, and oils.

The distribution of energy and nutrient intakes in our study revealed highest intake in overweight individuals. This result may be due to the quantitative increase in food consumption or qualitative changes in diet related to the increased consumption of high-energy foods. Additionally, a study of the US population showed that usual intakes differed significantly by body weight status and that dietary intakes of most nutrients were lowest in obese adults followed by overweight and normal weight adults, which were related to poor dietary choices among obese adults [41]. Moreover, some degree of underreporting may be present in our study. Overweight and obese subjects tend to underreport energy intake to a greater extent than normal-weight subjects [42]. In our study, mean energy and nutrient intakes were lower in obese individuals than in the other BMI categories, which is incompatible with the obese BMI.

Physical activity is a key component of weight management. Regular physical activity increases energy expenditure leading to an increase in caloric intake facilitating a varied diet and providing higher nutrient values [43]. Antioxidant nutrients, especially vitamin C, can help reduce the damage caused by free radicals, preventing injuries and improving physical performance [44]. Thus, the high levels of vitamin C found in physically active individuals can be explained by their high intake of fruit and vegetables. In addition, a highly skewed distribution of vitamin C was found in physically inactive individuals, which was due to high intake of vitamin C-rich foods by the top 5% of physically inactive individuals.

A strength of our study was the assessment of the total usual intakes of energy, nutrients, and food groups in an adult urban population using the ISU method. The estimates of usual energy and nutrient intake distributions were accurately adjusted for within-person variation, which corrects for any undesirable variation in nutrient intake distributions. The corrections in the data allowed the under or over reporting to be accommodate within the distribution of energy, nutrients, or food groups. A limitation of the study was the cross-sectional data, which prevents drawing directional conclusions or inferences about the causal relationships between BMI, physical activity, and dietary intake. Another limitation was adapting food categories in NDSR to meet the 2006 Brazilian National Dietary Guidelines, which may have resulted in the loss of some food items in the analysis.

## 5. Conclusions

This study described the usual intakes of energy, nutrients, and food groups along with the prevalence of inadequate or excessive micronutrient intake in an adult urban population. We found that the population consume large quantities of meat, beans, fat/oils, and exhibit a low prevalence of nutrient inadequacies, but have an excessive sodium intake. Energy and nutrient intakes are highest among men, in overweight and in physically active individuals. We also provided extensive material about nutrients distributions. This information is useful to a broader scientific audience to follow the distribution of nutrient intake in the population. Identification of intake within a certain percent level is informative. For example, if a study is conducted to evaluate a group of recreational female cyclists and obtained a mean intake of 300 mg of vitamin C, this value can be contrasted to the intake of women in the general population, which corresponds to the 75 to 90 percentiles of the intake distribution of the present study. Moreover, this study facilitates the development of interventions to reduce nutrient inadequacies and excessive consumption of food items associated with obesity and other chronic illness, which are major challenges for public health policy makers in Brazil and around the world.

## Figures and Tables

**Table 1:** Sociodemographic characteristics and percentage of energy provided by macronutrients among adults in an urban population. Brazil, 2016–2017.

Characteristics	*n*	%
Gender		
Male	216	43
Female	290	57
Age (years)		
20–30	187	37
31–50	175	35
50+	144	28
Education level		
Primary and High School	66	13
Bachelor or equivalent level	245	48
Master, Doctoral, or equivalent level	195	39
Socioeconomic level ^1^		
High	111	22
Medium	272	54
Low	123	24
Physical Activity ^2^		
No	119	24
Yes	387	76
BMI (kg/m²)		
<25	267	53
25–29.9	166	33
≥30	73	14
**Macronutrients**	**Mean**	**SD ^3^**
Male		
Energy (Kcal)	2373	615.3
Protein (%Kcal)	21	41.1
Carbohydrate (%Kcal)	44	80
Lipids (%Kcal)	32	24.9
Female		
Energy (Kcal)	1699	335.1
Protein (%Kcal)	18	16.3
Carbohydrate (%Kcal)	49	52.7
Lipids (%Kcal)	32	14.5

^1^, Monthly income: High: R$ 11,037 Brazilian Reais; Medium: R$ 4,562 Brazilian Reais; Low: R$ 1,345 Brazilian Reais; ^2^, No: <150 min per week of physical activity; Yes: >150 min per week of physical activity; ^3^, Standard Deviation.

**Table 2:** Selected results from the mean distribution and percentiles for usual intake, prevalence of inadequate intake, and percentage of individuals above tolerable upper intake levels value by gender and age. Brazil, 2016–2017.

Age (years)	*N*	EAR/AI ^1^	UL ^2^	Mean	SD ^3^	P25	SE ^4^	P50	SE	P75	SE	PI(%) ^5^	Above UL (%)
**Calcium (mg)**													
**Female**													
50+	88	1000	2000	1142.6	308.3	922.0	48.5	1111.0	43.5	1329.0	63.9	34.9	1.0
**Zinc (mg)**													
**Male**													
31–50+	140	9.4	40	14.6	6.2	10.4	0.8	13.4	0.9	17.5	1.5	17.4	0.8
**Female**													
31–50+	179	6.8	40	10.0	3.2	7.6	0.5	9.5	0.5	11.8	0.8	15.7	0.0
**Iron (mg)**													
**Female**													
20–50	202	8.1 *	45	11.6	1.6	10.8	-	11.7	-	12.4	-	11.4	0.0
**Vitamin A (mcg)**													
**Male**													
20–30	76	625	3000	1202.7	604.9	772.0	107.0	1083.0	116.0	1500.0	192.0	14.0	1.4
**Vitamin C (mcg)**													
**Male**													
20–50+	216	75	2000	208.4	314.1	78.5	14.4	128.5	21.1	226.0	53.4	23.0	0.6
**Female**													
20–50+	290	60	2000	283.1	862.3	64.5	10.8	113.0	19.0	231.0	63.3	22.2	1.7
**Vitamin D (mcg)**													
**Male**													
20–50+	216	10	100	5.8	2.6	4.0	0.6	5.4	0.6	7.2	0.9	93.1	0.0
**Female**													
20–50+	290	10	100	3.8	1.8	2.4	0.3	3.5	0.3	4.8	0.5	99.3	0.0
**Vitamin E (mcg)**													
**Male**													
20–50+	216	12	1000^a^	8.2	2.9	6.1	0.5	7.7	0.5	9.8	0.8	89.1	-
**Female**													
20–50+	290	12	1000^a^	6.2	1.9	4.8	0.3	5.9	0.3	7.2	0.5	98.8	-
**Vitamin B12 (mcg)**													
**Female**													
31–50+	179	2	-	3.5	1.4	2.4	0.2	3.2	0.2	4.2	0.4	12.3	-
**Folate (mcg)**													
**Male**													
20–50+	216	400	1000^a^	411.5	119.4	326.3	23.6	396.0	22.4	480.0	33.8	23.1	-
**Female**													
20–50+	290	400	1000^a^	305.3	65.2	259.0	14.5	300.0	13.1	346.0	18.1	61.9	-
**Sodium (g)**													
**Male**													
20–50	160	1.5^b^	2.3	4.2	1.0	3.5	0.2	4.1	0.2	4.8	0.3	-	98.2
50+	56	1.3^b^	2.3	3.7	1.0	3.0	0.2	3.5	0.2	4.3	0.3	-	95.3
**Female**													
20–50	202	1.5^b^	2.3	2.9	0.6	2.5	0.1	2.9	0.1	3.3	0.2	-	86.6
50+	88	1.3^b^	2.3	2.7	0.6	2.2	0.1	2.6	0.1	3.1	0.2	-	70.4
**Manganese (mg)**													
**Male**													
20–50+	216	2.3^b^	11	25.7	152.0	3.4	0.4	5.5	1.0	12.2	4.3	-	25.9
**Female**													
20–50+	290	1.8^b^	11	9.3	34.7	2.7	0.3	3.7	0.5	6.4	1.8	-	12.8

^1^, Estimated Average Requirements/Adequate Intake; ^2^, Tolerable Upper Intake Levels; ^3^, Standard Deviation; ^4^, Standard Error; ^5^, Prevalence of inadequacy; *, Inadequate iron intake was calculated using the probabilistic approach. ^a^, The Tolerable Upper Intake Levels (UL) for vitamin E, niacin, and folate apply to synthetic forms obtained from supplements, fortified foods, or a combination of the two; ^b^, Adequate Intake (AI).

**Table 3:** Usual intake of food groups according to the number of servings recommended by the 2006 Brazilian National Dietary Guidelines.

Food Group	Gender			BMI (kg/m^2^)			*p*-Value *	Physical Activity		*p*-Value *
	Male	Female	All	<25	25–29.9	≥30		Yes	No	
**Cereals**										
Mean	3.8	3.2	3.5	3.6	3.4	3.2		3.5	3.4	
SD ^1^	1.5	1.1	1.3	1.4	1.2	0.9		1.3	1.2	
% below six servings	91.7	98.5	95.8	94.4	96.7	99.4	0.13	95.6	96.9	0.62
**Vegetables**										
Mean	2.8	2.5	2.6	2.7	2.8	-		2.8	2.2	
SD	1.8	1.2	1.4	1.3	1.7	-		1.4	1.2	
% below three servings	66.4	72.1	74.1	64.9	67.3	-	0.72	66.7	80.2	0.03
**Fruits**										
Mean	4.3	3.4	3.8	4.1	3.9	2.9		4.2	2.8	
SD	2.7	2.6	2.8	3.0	2.0	3.2		3.3	3.4	
% below three servings	36.2	55.6	49.4	45.0	37.5	66.8	0.09	45.5	70.6	<0.001
**Meat**										
Mean	3.2	1.8	2.4	2.2	2.7	2.2		2.5	2.2	
SD	1.5	0.6	1.1	1.0	1.4	0.5		1.2	0.7	
% below one serving	1.3	3.2	2.8	4.9	3.5	0.0	0.96	4.6	1.4	0.18
**Dairy Products**										
Mean	2.1	1.5	1.8	1.7	1.9	1.8		1.8	1.8	
SD	1.0	0.6	0.9	0.8	0.9	1.0		0.9	0.7	
% below three servings	81.6	97.7	87.5	93.5	88.7	87.4	0.32	90.3	93.8	0.36
**Pulses**										
Mean	2.4	1.9	2.1	2.2	2.2	-		2.3	1.8	
SD	1.1	0.6	0.9	0.7	1.0	-		0.9	0.8	
% below one serving	5.6	2.6	6.5	1.8	5.3	-	0.18	3.2	16.3	<0.01
**Fat and Oils**										
Mean	2.6	2.1	2.3	2.3	2.5	1.8		2.3	2.2	
SD	0.6	0.6	0.7	0.7	0.8	0.1		0.6	0.8	
% below one serving	0.0	1.0	0.4	0.4	0.3	0.0	0.83	0.3	1.1	0.49
**Sugars**										
Mean	1.2	1.2	1.2	1.1	1.3	1.3		1.1	1.5	
SD	0.5	0.6	0.5	0.4	0.6	0.5		0.4	0.8	
% below one serving	40.8	43.3	41.7	50.2	34.7	33.3	0.02	47.8	32.3	0.02

^1^, Standard Deviation; *, Chi-square test.

## Supplementary Materials

The following are available online at http://www.mdpi.com/2072-6643/10/11/1714/s1, Table S1: Distribution of mean and percentiles of usual intake, prevalence of inadequate intake, and percentage of individuals above UL value by gender and age. Brazil, 2016–2017, Table S2: Distribution of mean and percentiles of usual intake of energy and macronutrients according to gender and age. Brazil, 2016-2017, Table S3: Distribution of means and percentiles of usual micronutrients intake according to BMI and physical activity. Brazil, 2016–2017, Table S4: Distribution of means and percentiles of usual intake of energy and macronutrients according to BMI and physical activity. Brazil, 2016–2017, Table S5: Distribution of mean and percentiles of usual grams of food and beverages intake according to gender, age, BMI and physical activity. Brazil, 2016–2017.
