# Supplementary material for: Assessment of Nutrient and Food Group Intakes across Sex, Physical Activity, and Body Mass Index in an Urban Brazilian Population
Source: Nutrients. 2018 Nov 9;10(11):1714. doi: 10.3390/nu10111714 (PMC6265884; doi:10.3390/nu10111714)
Supplement: Supplementary file 1 [file nutrients-10-01714-s001.pdf]

Supplementary Materials

Table S1. Distribution of mean and percentiles of usual intake, prevalence of inadequate intake, and percentage of individuals above UL value by gender and age. Brazil, 2016-2017

Age (years)	*N*	EAR/AI[1]	UL[2]	Mean	SD[3]	P5	SE[4]	P10	SE	P25	SE	P50	SE	P75	SE	P90	SE	P95	SE	Prevalence of inadequacy (%)	Above UL (%)
**Calcium (mg)**																					
**Male**																					
20-30	76	800	2500	1543.0	374.8	996.0	107.0	1094.0	96.7	1276.0	78.7	1506.0	72.4	1770.0	104.0	2039.0	162.0	2215.0	207.0	0.7	1.4
31-50	84	800	2500	1476.7	414.8	879.0	97.5	984.0	88.1	1180.0	71.8	1432.0	66.1	1725.0	97.3	2027.0	155.0	2226.0	200.0	2.6	1.7
50+	56	800	2000	1479.7	402.4	860.0	144.0	980.0	126.0	1196.0	96.4	1456.0	82.5	1738.0	114.0	2010.0	171.0	2180.0	212.0	3.3	10.4
**Female**																					
20-30	111	800	2500	1197.9	326.5	702.0	76.7	795.0	68.5	966.0	54.3	1175.0	47.7	1405.0	65.2	1629.0	98.1	1771.0	123.0	10.3	0.1
31-50	91	800	2500	1174.6	245.7	810.0	80.2	878.0	71.9	1000.0	58.0	1153.0	52.6	1326.0	73.3	1499.0	113.0	1611.0	143.0	4.4	0.0
50+	88	1000	2000	1142.6	308.3	695.0	66.4	774.0	60.1	922.0	48.5	1111.0	43.5	1329.0	63.9	1550.0	102.0	1696.0	132.0	34.9	1.0
**All**	506	-	-	1317.0	401.6	745.0	35.9	843.0	32.8	1029.0	27.3	1271.0	25.5	1555.0	37.6	1850.0	60.2	2046.0	77.9	-	-
**Phosphorous (mg)**																					
**Male**																					
20-30	76	580	4000	1791.5	443.6	1108.0	135.0	1241.0	119.0	1479.0	92.6	1766.0	79.9	2076.0	108.0	2375.0	161.0	2562.0	200.0	0.0	0.0
31-50	84	580	4000	1611.7	543.9	876.0	99.8	993.0	92.5	1222.0	78.4	1532.0	75.5	1915.0	119.0	2332.0	203.0	2619.0	271.0	0.3	0.1
50+	56	580	4000	1561.7	470.5	915.0	123.0	1021.0	113.0	1225.0	95.0	1498.0	90.5	1828.0	140.0	2184.0	234.0	2427.0	310.0	0.1	0.0
**Female**																					
20-30	111	580	4000	1141.1	125.8	940.0	67.7	982.0	58.2	1054.0	44.0	1138.0	37.1	1224.0	47.2	1304.0	67.0	1354.0	81.5	0.0	0.0
31-50	91	580	4000	1082.9	215.6	756.0	75.6	819.0	66.7	931.0	52.7	1068.0	47.0	1219.0	64.2	1367.0	96.2	1461.0	120.0	0.3	0.0
50+	88	580	4000	1093.2	259.3	725.0	58.1	789.0	52.6	908.0	43.1	1063.0	40.0	1245.0	59.4	1435.0	96.5	1563.0	126.0	0.5	0.0
**All**	506	-	-	1347.9	440.4	749.0	34.2	845.0	31.8	1033.0	27.2	1285.0	26.4	1594.0	40.8	1930.0	68.3	2160.0	91.0	-	-
**Magnesium (mg)**																					
**Male**																					
20-30	76	330	350[a]	607.3	151.8	391.0	35.8	429.0	32.4	499.0	26.5	590.0	24.6	697.0	36.5	808.0	59.0	882.0	76.9	1.0	-
31-50	84	350	350[a]	550.8	134.3	365.0	27.8	396.0	25.3	455.0	20.9	533.0	19.7	627.0	30.4	728.0	50.8	797.0	67.5	3.3	-
50+	56	350	350[a]	555.1	145.0	336.0	45.1	377.0	39.4	452.0	30.1	545.0	25.8	647.0	37.0	747.0	57.2	810.0	71.9	6.5	-
**Female**																					
20-30	111	255	350[a]	441.6	71.5	326.0	25.5	351.0	21.8	393.0	16.2	441.0	13.3	489.0	16.8	534.0	23.5	561.0	28.4	0.3	-
31-50	91	265	350[a]	455.2	111.5	287.0	28.8	319.0	25.5	376.0	20.1	447.0	17.8	525.0	24.7	602.0	37.8	652.0	47.6	2.8	-
50+	88	265	350[a]	432.2	91.3	285.0	28.0	316.0	23.9	369.0	17.5	430.0	14.2	493.0	18.4	550.0	26.5	585.0	32.3	2.9	-
**All**	506	-	-	498.2	136.5	300.0	12.2	335.0	11.0	401.0	9.0	484.0	8.2	580.0	11.9	679.0	19.0	744.0	24.4	-	-

Table S1. Continued.

Age (years)	*N*	EAR/AI[1]	UL[2]	Mean	SD[3]	P5	SE[4]	P10	SE	P25	SE	P50	SE	P75	SE	P90	SE	P95	SE	Prevalence of inadequacy (%)	Above UL (%)
												**Zinc (mg)**									
**Male**																					
20-30	76	9.4	40	17.8	2.6	13.8	1.7	14.6	1.5	16.0	1.2	17.6	1.0	19.5	1.4	21.2	2.0	22.3	2.5	0.0	0.0
31-50	84	9.4	40	15.6	7.9	6.7	0.9	7.8	0.9	10.1	0.8	13.7	0.9	18.9	1.7	25.5	3.3	30.6	4.9	20.0	1.5
50+	56	9.4	40	13.7	4.4	7.8	1.1	8.7	1.0	10.6	0.9	13.1	0.8	16.1	1.3	19.5	2.2	21.8	3.0	14.8	0.0
**Female**																					
20-30	111	6.8	40	10.2	2.1	7.1	0.6	7.7	0.6	8.7	0.5	10.0	0.4	11.4	0.6	12.9	0.9	13.9	1.2	3.0	0.0
31-50	91	6.8	40	10.0	3.2	5.4	0.7	6.2	0.6	7.6	0.5	9.5	0.5	11.8	0.8	14.3	1.3	15.9	1.7	15.7	0.0
50+	88	6.8	40	9.3	1.0	7.7	0.7	8.0	0.6	8.5	0.5	9.2	0.4	9.9	0.6	10.6	0.9	11.1	1.1	0.3	0.0
**All**	506	-	-	12.5	4.9	6.3	0.3	7.2	0.3	9.0	0.3	11.6	0.3	14.9	0.5	18.8	0.9	21.6	1.2	-	-
												**Copper (mg)**									
**Male**																					
20-30	76	700	10000	1423.6	404.1	830.0	106.0	937.0	95.5	1135.0	77.2	1386.0	70.1	1671.0	100.0	1959.0	156.0	2147.0	199.0	1.7	0.0
31-50	84	700	10000	1504.4	502.5	826.0	105.0	934.0	98.0	1145.0	84.5	1431.0	82.9	1783.0	129.0	2168.0	216.0	2434.0	289.0	1.7	0.0
50+	56	700	10000	1574.5	677.7	880.0	110.0	975.0	101.0	1153.0	85.0	1410.0	91.3	1796.0	182.0	2348.0	414.0	2819.0	655.0	0.8	0.0
**Female**																					
20-30	111	700	10000	1197.1	282.7	895.0	68.0	933.0	66.8	1011.0	64.7	1132.0	73.5	1303.0	132.0	1532.0	273.0	1719.0	405.0	0.0	0.0
31-50	91	700	10000	1123.1	369.4	623.0	72.8	703.0	67.9	859.0	58.6	1069.0	57.4	1328.0	88.6	1611.0	148.0	1806.0	198.0	9.8	0.0
50+	88	700	10000	1084.6	214.5	772.0	77.5	828.0	69.6	932.0	56.5	1064.0	51.9	1214.0	74.0	1368.0	116.0	1469.0	149.0	1.6	0.0
**All**	506	-	-	1330.4	579.5	751.0	35.0	834.0	31.7	990.0	26.6	1206.0	28.6	1510.0	52.5	1924.0	110.0	2288.0	186.0	-	-
												**Selenium (mcg)**									
**Male**																					
20-30	76	45	400	190.9	55.0	118.0	14.5	130.0	13.2	153.0	10.9	182.0	10.6	220.0	16.7	262.0	29.6	292.0	40.7	0.0	0.4
31-50	84	45	400	176.9	85.0	74	11.5	88.0	11.3	117.0	10.4	160.0	11.0	218.0	19.0	286.0	35.3	337.0	49.9	0.3	2.1
50+	56	45	400	156.1	65.5	84.0	10.2	92.0	9.9	111.0	9.3	141.0	10.6	184.0	19.9	238.0	39.4	281.0	57.8	0.0	0.8
**Female**																					
20-30	111	45	400	121.1	2.3	117.3	11.8	118.2	10.0	119.5	7.4	121.1	6.2	122.7	7.7	124.2	10.6	125.0	12.6	0.0	0.0
31-50	91	45	400	113.1	33.9	67.0	8.8	75.0	7.9	90.0	6.4	108.0	6.1	131.0	9.6	157.0	16.9	175.0	23.1	0.2	0.0
50+	88	45	400	111.0	49.2	62.0	5.8	69.0	5.4	81.0	4.7	99.0	5.2	126.0	10.5	166.0	24.1	201.0	38.5	0.3	0.3
**All**	506	-	-	141.9	58.4	73.0	4.0	83.0	3.6	102.0	3.2	130.0	3.4	168.0	6.1	216.0	11.4	252.0	16.4	-	-

Table S1. Continued.

Age (years)	*N*	EAR/AI[1]	UL[2]	Mean	SD[3]	P5	SE[4]	P10	SE	P25	SE	P50	SE	P75	SE	P90	SE	P95	SE	Prevalence of inadequacy (%)	Above UL (%)
												**Iron (mg)**									
**Male**																					
20-30	76	6	45	16.6	3.3	11.6	1.2	12.5	1.1	14.2	0.9	16.3	0.8	18.6	1.1	20.9	1.7	22.4	2.1	0.0	0.0
31-50	84	6	45	15.5	5.2	8.7	0.9	9.7	0.8	11.8	0.7	14.7	0.7	18.3	1.1	22.3	2.0	25.2	2.7	0.3	0.0
50+	56	6	45	14.7	3.8	9.8	0.9	10.5	0.8	12.0	0.7	14.0	0.7	16.7	1.1	19.7	2.0	21.8	2.8	0.0	0.0
**Female**																					
20-50	202	8.1*	45	11.6	1.6	8.6	-	9.6	-	10.8	-	11.7	-	12.4	-	13.3	-	14.1	-	11.4	0.0
50+	88	5*	45	10.7	0.9	9.1	-	9.5	-	10.1	-	11.3	-	12.1	-	12.4	-	13.5	-	0.0	0.0
**All**	506	-	-	13.2	3.8	8.1	0.3	9.0	0.3	10.6	0.2	12.7	0.2	15.3	0.4	18.2	0.6	20.2	0.9	-	-
												**Sodium (g)**									
**Male**																					
20-30	76	1.5[b]	2.3	4.4	0.8	3.2	0.3	3.4	0.3	3.8	0.2	4.3	0.2	4.9	0.3	5.4	0.4	5.7	0.5	-	99.9
31-50	84	1.5[b]	2.3	4.1	1.3	2.4	0.2	2.7	0.2	3.1	0.2	3.8	0.2	4.7	0.3	5.7	0.6	6.5	0.8	-	96.5
50+	56	1.3[b]	2.3	3.7	1.0	2.3	0.3	2.5	0.2	3.0	0.2	3.5	0.2	4.3	0.3	5.1	0.6	5.7	0.8	-	95.3
**Female**																					
20-30	111	1.5[b]	2.3	3.0	0.5	2.2	0.2	2.4	0.2	2.7	0.1	3.0	0.1	3.4	0.2	3.7	0.2	4.0	0.3	-	92.1
31-50	91	1.5[b]	2.3	2.8	0.6	2.0	0.2	2.1	0.2	2.4	0.1	2.8	0.1	3.2	0.2	3.6	0.3	3.9	0.4	-	81.1
50+	88	1.3[b]	2.3	2.7	0.6	1.8	0.2	1.9	0.1	2.2	0.1	2.6	0.1	3.1	0.2	3.5	0.3	3.8	0.3	-	70.4
**All**	506	-	-	3.4	1.0	1.9	0.1	2.2	0.1	2.6	0.1	3.2	0.1	4.0	0.1	4.8	0.2	5.3	0.2	-	-
												**Potassium (mg)**									
**Male**																					
20-30	76	4.7[b]	-	3.4	0.8	2.2	0.3	2.4	0.2	2.9	0.2	3.4	0.2	3.9	0.2	4.5	0.3	4.8	0.4	-	-
31-50	84	4.7[b]	-	3.2	0.8	2.0	0.2	2.2	0.2	2.6	0.1	3.1	0.1	3.7	0.2	4.3	0.3	4.6	0.4	-	-
50+	56	4.7[b]	-	3.5	0.8	2.3	0.3	2.5	0.3	2.9	0.2	3.4	0.2	4.0	0.3	4.6	0.4	5.0	0.5	-	-
**Female**																					
20-30	111	4.7[b]	-	2.3	0.4	1.7	0.1	1.8	0.1	2.1	0.1	2.3	0.1	2.6	0.1	2.9	0.2	3.0	0.2	-	-
31-50	91	4.7[b]	-	2.4	0.7	1.4	0.2	1.6	0.1	1.9	0.1	2.4	0.1	2.8	0.2	3.3	0.2	3.7	0.3	-	-
50+	88	4.7[b]	-	2.5	0.5	1.7	0.1	1.9	0.1	2.1	0.1	2.5	0.1	2.9	0.1	3.3	0.2	3.5	0.3	-	-
**All**	506	-	-	2.8	0.8	1.7	0.1	1.9	0.1	2.2	0.1	2.7	0.1	3.3	0.1	3.9	0.1	4.3	0.2	-	-

Table S1. Continued.

Age (years)	*N*	EAR/AI[1]	UL[2]	Mean	SD[3]	P5	SE[4]	P10	SE	P25	SE	P50	SE	P75	SE	P90	SE	P95	SE	Prevalence of inadequacy (%)	Above UL (%)
												**Manganese (mg)**									
**Male**																					
20-30	76	2.3[b]	11	8.9	11.5	2.5	0.4	2.9	0.5	3.8	0.5	5.6	0.9	9.5	2.4	17.1	6.8	25.1	12.7	-	20.3
31-50	84	2.3[b]	11	49.3	366.5	1.9	0.3	2.2	0.3	3.1	0.4	5.5	1.1	15.7	6.2	58.5	38.1	138.5	113.0	-	31.5
50+	56	2.3[b]	11	18.8	78.0	2.3	0.4	2.6	0.3	3.4	0.4	5.3	1.0	11.5	4.4	31.3	20.2	61.4	51.0	-	26.1
**Female**																					
20-30	111	1.8[b]	11	15.2	79.0	2.0	0.2	2.2	0.2	2.6	0.2	3.8	0.5	7.9	2.3	22.0	12.1	45.5	32.9	-	18.6
31-50	91	1.8[b]	11	5.4	13.8	1.5	0.2	1.7	0.2	2.2	0.2	3.0	0.2	4.5	0.7	8.5	3.0	14.3	7.0	-	7.1
50+	88	1.8[b]	11	7.1	11.5	2.5	0.3	2.7	0.3	3.1	0.4	4.2	0.7	6.9	2.4	12.9	8.0	20.0	16.3	-	12.7
**All**	506	-	-	10.5	14.5	1.9	0.1	2.2	0.1	2.8	0.1	4.2	0.3	12.1	2.1	28.8	3.2	37.9	4.2	-	-
												**Vitamin A (mcg)**									
**Male**																					
20-30	76	625	3000	1202.7	604.9	463.0	112.0	563.0	112.0	772.0	107.0	1083.0	116.0	1500.0	192.0	1991.0	342.0	2349.0	474.0	14.0	1.4
31-50	84	625	3000	1066.5	267.4	693.0	108.0	757.0	97.8	878.0	80.8	1034.0	76.2	1218.0	113.0	1416.0	188.0	1552.0	252.0	1.9	0.0
50+	56	625	3000	1796.0	844.3	880.0	156.0	986.0	153.0	1219.0	148.0	1593.0	173.0	2140.0	325.0	2846.0	650.0	3398.0	961.0	0.2	8.2
**Female**																					
20-30	111	500	3000	1110.6	548.6	504.0	88.1	583.0	85.7	745.0	80.1	987.0	89.3	1331.0	160.0	1776.0	319.0	2131.0	476.0	4.8	1.1
31-50	91	500	3000	1061.1	597.2	435.0	82.4	513.0	80.7	675.0	75.4	918.0	84.8	1276.0	158.0	1761.0	335.0	2162.0	515.0	9.0	1.4
50+	88	500	3000	1186.2	583.7	501.0	78.2	590.0	77.0	779.0	72.6	1062.0	78.9	1453.0	138.0	1931.0	260.0	2290.0	372.0	4.9	1.3
**All**	506	-	-	1215.6	643.1	505.0	41.1	599.0	39.8	789.0	36.4	1070.0	40.0	1470.0	72.5	1993.0	148.0	2412.0	222.0	-	-
												**Vitamin C (mg)**									
**Male**																					
20-30	76	75	2000	141.0	97.8	40.0	12.6	51.0	13.1	76.0	13.5	116.0	16.2	177.0	30.6	259.0	62.6	325.0	94.1	24.4	0.0
31-50	84	75	2000	275.8	530.5	43.0	11.6	54.0	12.3	81.0	15.2	141.0	25.9	275.0	76.1	556.0	244.0	883.0	493.0	21.6	1.2
50+	56	75	2000	841.8	867.3	98.0	75.6	149.0	100.0	297.0	128.0	577.0	174.0	1073.0	396.0	1834.0	892.0	2485.0	1380.0	2.8	8.3
**Female**																					
20-30	111	60	2000	267.3	637.6	33.0	8.2	42.0	9.0	65.0	11.7	117.0	21.4	243.0	68.1	537.0	237.0	899.0	500.0	22.0	1.5
31-50	91	60	2000	299.0	1087.0	32.0	8.9	41.0	9.0	64.0	9.9	109.0	16.6	219.0	58.4	512.0	243.0	939.0	586.0	22.4	1.9
50+	88	60	2000	171.8	165.8	52.0	13.9	66.0	13.2	92.0	10.8	130.0	12.3	194.0	29.4	304.0	79.1	417.0	144.0	7.6	0.1
**All**	506	-	-	246.6	341.2	37.0	3.9	47.0	4.1	70.0	4.8	126.0	10.1	287.0	35.4	580.0	77.3	828.0	126.0	-	-

Table S1. Continued.

Age (years)	*N*	EAR/AI[1]	UL[2]	Mean	SD[3]	P5	SE[4]	P10	SE	P25	SE	P50	SE	P75	SE	P90	SE	P95	SE	Prevalence of inadequacy (%)	Above UL (%)
**Vitamin D (mcg)**																					
**Male**																					
20-30	76	10	100	6.0	3.1	2.3	0.6	2.8	0.6	3.8	0.5	5.4	0.6	7.6	1.0	10.1	1.8	12.0	2.5	89.6	0.0
31-50	84	10	100	6.1	2.2	3.1	0.7	3.6	0.7	4.5	0.6	5.7	0.6	7.3	0.9	8.9	1.5	10.1	2.0	94.8	0.0
50+	56	10	100	5.4	2.5	2.2	0.6	2.7	0.6	3.6	0.6	5.0	0.6	6.7	0.9	8.7	1.6	10.0	2.1	94.9	0.0
**Female**																					
20-30	111	10	100	4.1	1.9	1.7	0.3	2.0	0.3	2.7	0.3	3.8	0.3	5.0	0.5	6.5	0.9	7.5	1.3	98.9	0.0
31-50	91	10	100	3.5	1.7	1.3	0.3	1.7	0.3	2.3	0.3	3.3	0.3	4.4	0.5	5.8	0.8	6.7	1.1	99.7	0.0
50+	88	10	100	3.8	2.0	1.2	0.3	1.5	0.3	2.3	0.3	3.4	0.3	4.9	0.5	6.4	0.8	7.4	1.1	99.2	0.0
**All**	506	-	-	4.8	2.6	1.7	0.2	2.1	0.2	2.9	0.2	4.2	0.2	6.0	0.3	8.1	0.6	9.7	0.8	-	-
**Vitamin E (mg)**																					
**Male**																					
20-30	76	12	1000[a]	8.7	3.5	4.0	0.7	4.8	0.6	6.2	0.5	8.2	0.5	10.6	0.8	13.4	1.4	15.2	1.9	83.9	-
31-50	84	12	1000[a]	8.6	3.0	4.7	0.6	5.3	0.6	6.5	0.5	8.1	0.5	10.2	0.8	12.5	1.4	14.1	2.0	87.7	-
50+	56	12	1000[a]	7.3	2.4	4.3	0.6	4.8	0.6	5.7	0.5	6.9	0.4	8.5	0.7	10.3	1.4	11.7	1.9	95.6	-
**Female**																					
20-30	111	12	1000[a]	6.3	1.5	4.1	0.4	4.5	0.4	5.2	0.3	6.1	0.3	7.2	0.5	8.3	0.7	9.1	0.9	99.8	-
31-50	91	12	1000[a]	6.3	2.1	3.5	0.5	3.9	0.4	4.8	0.4	6.0	0.4	7.4	0.6	9.0	1.0	10.1	1.3	98.5	-
50+	88	12	1000[a]	5.9	2.2	3.2	0.4	3.6	0.3	4.4	0.3	5.5	0.3	7.0	0.6	8.8	1.1	10.1	1.5	98.0	-
**All**	506	-	-	7.2	2.7	3.7	0.2	4.3	0.2	5.3	0.2	6.7	0.2	8.5	0.3	10.6	0.5	12.2	0.7	-	-
**Vitamin K (mcg)**																					
**Male**																					
20-30	76	120[b]	-	123.4	61.7	55.0	10.2	64.0	9.8	83.0	8.9	110.0	9.6	148.0	17.3	198.0	35.4	238.0	53.4	-	-
31-50	84	120[b]	-	126.2	42.5	70.0	11.6	78.0	10.9	96.0	9.6	120.0	9.6	149.0	14.8	182.0	25.0	205.0	33.7	-	-
50+	56	120[b]	-	119.1	27.0	79.0	13.5	86.0	12.1	100.0	9.7	117.0	8.7	136.0	12.2	155.0	18.8	167.0	24.0	-	-
**Female**																					
20-30	111	90[b]	-	118.4	46.8	59.0	9.8	68.0	9.4	85.0	8.6	110.0	9.1	142.0	14.7	179.0	25.8	206.0	35.5	-	-
31-50	91	90[b]	-	127.5	65.4	52.0	10.0	62.0	10.0	82.0	9.7	113.0	10.9	156.0	19.1	210.0	36.0	251.0	51.7	-	-
50+	88	90[b]	-	112.2	58.8	47.0	8.5	55.0	8.4	72.0	8.2	99.0	9.3	137.0	16.9	186.0	33.0	223.0	48.3	-	-
**All**	506	-	-	122.3	54.8	56.0	4.2	65.0	4.1	84.0	3.8	111.0	4.2	149.0	6.9	193.0	12.4	226.0	17.4	-	-

Table S1. Continued.

Age (years)	*N*	EAR/AI[1]	UL[2]	Mean	SD[3]	P5	SE[4]	P10	SE	P25	SE	P50	SE	P75	SE	P90	SE	P95	SE	Prevalence of inadequacy (%)	Above UL (%)
**Vitamin B1 (mg)**																					
**Male**																					
20-30	76	1	-	1.9	0.6	1.1	0.1	1.2	0.1	1.5	0.1	1.8	0.1	2.2	0.1	2.7	0.2	2.9	0.3	3.4	-
31-50	84	1	-	1.8	0.7	1.0	0.1	1.1	0.1	1.3	0.1	1.7	0.1	2.2	0.2	2.7	0.3	3.1	0.4	6.0	-
50+	56	1	-	1.8	0.4	1.2	0.1	1.3	0.1	1.5	0.1	1.7	0.1	2.0	0.2	2.3	0.3	2.6	0.3	0.7	-
**Female**																					
20-30	111	0.9	-	1.4	0.3	1.0	0.1	1.0	0.1	1.2	0.1	1.3	0.1	1.5	0.1	1.7	0.1	1.8	0.2	2.3	-
31-50	91	0.9	-	1.3	0.3	0.8	0.1	0.9	0.1	1.1	0.1	1.2	0.1	1.5	0.1	1.7	0.1	1.8	0.2	9.8	-
50+	88	0.9	-	1.3	0.1	1.1	0.1	1.1	0.1	1.2	0.1	1.3	0.0	1.4	0.1	1.5	0.1	1.5	0.1	0.1	-
**All**	506	-	-	1.5	0.5	0.9	0.0	1.0	0.0	1.2	0.0	1.5	0.0	1.8	0.0	2.2	0.1	2.4	0.1	-	-
**Vitamin B2 (mg)**																					
**Male**																					
20-30	76	1.1	-	2.1	0.4	1.4	0.2	1.5	0.1	1.8	0.1	2.0	0.1	2.3	0.1	2.6	0.2	2.8	0.2	0.6	-
31-50	84	1.1	-	1.9	0.6	1.1	0.1	1.2	0.1	1.5	0.1	1.9	0.1	2.3	0.1	2.8	0.2	3.1	0.3	6.3	-
50+	56	1.1	-	2.0	0.5	1.3	0.2	1.4	0.2	1.7	0.1	2.0	0.1	2.3	0.2	2.7	0.3	2.9	0.4	1.2	-
**Female**																					
20-30	111	0.9	-	1.5	0.2	1.2	0.1	1.3	0.1	1.4	0.1	1.5	0.1	1.6	0.1	1.7	0.1	1.8	0.1	0.0	-
31-50	91	0.9	-	1.4	0.2	1.0	0.1	1.1	0.1	1.2	0.1	1.4	0.1	1.6	0.1	1.7	0.1	1.8	0.1	1.1	-
50+	88	0.9	-	1.4	0.2	1.1	0.1	1.2	0.1	1.3	0.1	1.4	0.0	1.6	0.1	1.8	0.1	1.9	0.1	0.6	-
**All**	506	-	-	1.7	0.5	1.0	0.0	1.2	0.0	1.4	0.0	1.6	0.0	2.0	0.0	2.3	0.1	2.5	0.1	-	-
**Vitamin B3 (mg)**																					
**Male**																					
20-30	76	12	35[a]	39.8	7.9	28.1	3.7	30.2	3.3	34.2	2.7	39.1	2.4	44.6	3.4	50.2	5.2	53.8	6.6	0.0	-
31-50	84	12	35[a]	30.2	8.8	18.0	2.4	20.0	2.3	23.9	1.9	29.0	1.9	35.2	2.8	41.8	4.5	46.3	6.0	0.2	-
50+	56	12	35[a]	25.3	6.7	16.0	2.1	17.6	1.9	20.5	1.6	24.5	1.5	29.2	2.3	34.2	3.9	37.6	5.1	0.3	-
**Female**																					
20-30	111	11	35[a]	21.5	3.1	16.8	1.8	17.8	1.6	19.4	1.2	21.3	1.1	23.5	1.5	25.6	2.2	26.9	2.7	0.0	-
31-50	91	11	35[a]	19.0	4.3	12.5	1.4	13.7	1.3	15.9	1.0	18.6	0.9	21.7	1.3	24.7	2.0	26.7	2.5	1.6	-
50+	88	11	35[a]	18.0	0.7	16.9	1.5	17.1	1.2	17.5	0.9	18.0	0.8	18.5	1.0	19.0	1.4	19.3	1.6	0.0	-
**All**	506	-	-	25.3	8.8	13.8	0.7	15.6	0.7	19.0	0.6	23.9	0.6	30.0	1.0	36.8	1.7	41.6	2.3	-	-

Table S1. Continued.

Age (years)	*N*	EAR/AI[1]	UL[2]	Mean	SD[3]	P5	SE[4]	P10	SE	P25	SE	P50	SE	P75	SE	P90	SE	P95	SE	Prevalence of inadequacy (%)	Above UL (%)
												**Vitamin B5 (mg)**									
**Male**																					
20-30	76	5[b]	-	7.1	1.7	4.6	0.5	5.1	0.4	5.9	0.4	6.9	0.3	8.1	0.5	9.3	0.7	10.1	0.9	-	-
31-50	84	5[b]	-	6.3	1.8	3.8	0.4	4.2	0.4	5.0	0.3	6.1	0.3	7.4	0.4	8.7	0.7	9.5	0.9	-	-
50+	56	5[b]	-	6.3	1.4	4.2	0.5	4.6	0.5	5.3	0.4	6.1	0.3	7.1	0.5	8.1	0.8	8.8	1.0	-	-
**Female**			-																		
20-30	111	5[b]	-	4.8	0.4	4.2	0.3	4.3	0.3	4.5	0.2	4.8	0.2	5.1	0.2	5.4	0.3	5.5	0.4	-	-
31-50	91	5[b]	-	4.3	0.9	3.0	0.3	3.2	0.2	3.7	0.2	4.3	0.2	4.9	0.2	5.6	0.4	6.0	0.5	-	-
50+	88	5[b]	-	4.7	0.8	3.6	0.2	3.8	0.2	4.2	0.2	4.6	0.1	5.2	0.2	5.7	0.3	6.1	0.4	-	-
**All**	506	-	-	5.5	1.5	3.4	0.1	3.7	0.1	4.4	0.1	5.3	0.1	6.3	0.2	7.5	0.3	8.3	0.3	-	-
												**Vitamin B6 (mg)**									
**Male**																					
20-30	76	1.1	100	3.0	0.9	1.8	0.2	2.0	0.2	2.4	0.2	3.0	0.2	3.6	0.2	4.2	0.4	4.6	0.5	0.1	0
31-50	84	1.1	100	2.3	0.7	1.4	0.2	1.5	0.2	1.8	0.1	2.2	0.1	2.7	0.2	3.3	0.3	3.6	0.4	1.1	0
50+	56	1.4	100	2.2	0.6	1.4	0.2	1.5	0.2	2.1	0.1	2.1	0.1	2.5	0.2	3.0	0.3	3.3	0.4	6.5	0
**Female**																					
20-30	111	1.1	100	1.7	0.3	1.3	1.3	1.4	0.1	1.5	0.1	1.7	0.1	1.9	0.1	2.1	0.2	2.2	0.2	0.5	0
31-50	91	1.1	100	1.6	0.4	1.0	0.1	1.1	0.1	1.3	0.1	1.5	0.1	1.8	0.1	2.1	0.2	2.3	0.2	10.9	0
50+	88	1.3	100	1.6	0.1	1.5	0.1	1.5	0.1	1.5	0.1	1.6	0.1	1.6	0.1	1.7	0.1	1.7	0.1	0.0	0
**All**	506	-	-	2.0	0.7	1.1	0.1	1.3	0.1	1.5	0.0	1.9	0.0	2.4	0.1	2.9	0.1	3.3	0.2	-	-
												**Vitamin B12 (mcg)**									
**Male**																					
20-30	76	2	-	6.4	1.5	4.1	0.7	5.3	0.5	6.2	0.4	7.3	0.6	7.3	0.6	8.3	1.0	9.0	1.3	0.0	-
31-50	84	2	-	5.4	2.5	2.1	0.4	2.6	0.4	3.6	0.4	4.9	0.4	6.7	0.6	8.7	1.1	10.1	1.5	3.9	-
50+	56	2	-	6.8	4.4	2.9	0.6	3.3	0.6	4.1	0.6	5.5	0.8	7.9	1.7	11.4	4.0	14.6	6.6	0.2	-
**Female**																					
20-30	111	2	-	4.7	3.6	2.1	0.3	2.3	0.2	2.8	0.2	3.7	0.3	5.2	0.8	7.8	1.9	10.3	3.4	3.9	-
31-50	91	2	-	3.8	1.8	1.6	0.3	1.9	0.2	2.5	0.2	3.4	0.3	4.7	0.4	6.0	0.7	7.0	1.1	11.8	-
50+	88	2	-	3.2	1.1	1.6	0.3	1.9	0.3	2.4	0.2	3.1	0.2	3.8	0.3	4.6	0.4	5.1	0.6	12.7	-
**All**	506	-	-	5.0	3.5	1.9	0.1	2.3	0.1	3.0	0.1	4.1	0.1	5.7	0.3	8.5	0.8	11.1	1.3	-	-

Table S1. Continued.

Age (years)	*N*	EAR/AI[1]	UL[2]	Mean	SD[3]	P5	SE[4]	P10	SE	P25	SE	P50	SE	P75	SE	P90	SE	P95	SE	Prevalence of inadequacy (%)	Above UL (%)
												**Folate (mcg)**									
**Male**																					
20-30	76	400	1000[a]	403.8	130.7	228.0	27.8	256.0	25.9	310.0	22.4	384.0	22.1	476.0	34.5	576.0	58.3	646.0	78.0	28.2	-
31-50	84	400	1000[a]	394.3	93.7	256.0	29.3	281.0	26.3	328.0	21.2	386.0	19.2	452.0	27.2	518.0	42.1	561.0	53.7	22.1	-
50+	56	400	1000[a]	436.4	133.7	252.0	34.9	282.0	32.3	341.0	27.3	418.0	25.9	512.0	39.6	613.0	66.1	682.0	88.0	18.9	-
**Female**																					
20-30	111	400	1000[a]	310.2	76.8	198.0	19.5	218.0	17.7	255.0	14.5	303.0	13.4	357.0	19.1	412.0	29.6	448.0	37.9	58.9	-
31-50	91	400	1000[a]	297.2	71.3	191.0	20.6	210.0	18.3	247.0	14.7	291.0	13.2	342.0	18.3	391.0	27.9	423.0	35.2	65.3	-
50+	88	400	1000[a]	308.6	47.5	236.0	21.2	250.0	18.6	275.0	14.4	306.0	12.6	339.0	16.9	371.0	25.2	391.0	31.4	61.6	-
**All**	506	-	-	350.8	105.0	205.0	9.6	229.0	8.9	276.0	7.6	337.0	7.3	411.0	11.0	490.0	18.0	543.0	23.7	-	-

[1]Estimated Avarage Requirements/ Adequate Intake

[2]Tolerable Upper Intake Levels

[3]Standard Deviation

[4]Standard Error

[a]The ULs for vitamin E, niacin, and folate apply to synthetic forms obtained from supplements, fortified foods, or a combination of the two.

[b]Adequate Intake (AI)

*Inadequate iron intake was calculated using the probabilistic approach

Table S2. Distribution of mean and percentiles of usual intake of energy and macronutrients according to gender and age. Brazil, 2016-2017.

Age (years)	*N*	Mean	SD[1]	P5	SE[2]	P10	SE	P25	SE	P50	SE	P75	SE	P90	SE	P95	SE
**Energy (kcal)**																	
**Male**																	
20-30	76	2589	476.7	1847	177	1995	154	2256	118	2566	102	2897	136	3214	199	3412	244
31-50	84	2390	727.6	1380	139	1548	128	1869	106	2295	98.5	2808	153	3354	256	3724	339
50+	56	2008	343.8	1516	141	1605	115	1761	86.3	1965	87.2	2222	137	2485	197	2645	221
**Female**																	
20-30	111	1753	210.4	1419	93.8	1488	81	1607	61.6	1746	52.2	1891	67	2026	95.8	2110	117
31-50	91	1699	398.0	1108	107	1219	94.5	1419	74	1665	65.7	1941	93.1	2221	147	2406	189
50+	88	1618	304.9	1156	89.4	1244	78.9	1402	61.9	1596	54.5	1809	75.4	2019	115	2153	144
**All**	506	1984	556.1	1203	48.7	1336	43.6	1585	35.7	1913	34.3	2310	52.3	2729	83.4	3006	106
**Carbohydrate (g)**																	
**Male**																	
20-30	76	269	72.8	162	19.6	181	17.6	217	14.3	262	13.1	313	18.9	365	29.5	399	37.4
31-50	84	267	97.5	122	22.1	148	20.1	197	16.1	259	14.2	328	20.3	397	31.6	441	40
50+	56	251	61.8	162	19.1	178	17.3	207	14.1	244	13.0	287	19.2	332	31	363	40.4
**Female**																	
20-30	111	211	44.0	144	12.8	157	11.4	180	9.07	207	8.1	238	11.1	269	16.7	288	20.9
31-50	91	208	61.0	121	15.6	137	13.6	166	10	201	8.7	241	13.3	285	23.9	317	32.8
50+	88	206	46.3	137	12.9	150	11.6	173	9.29	202	8.4	234	11.8	267	18.3	288	23.3
**All**	506	233	71.1	132	6.8	150	6.12	183	4.88	224	4.5	273	6.68	325	11.2	361	14.9

Table S2. Continued.

Age (years)	*N*	Mean	SD[1]	P5	SE[2]	P10	SE	P25	SE	P50	SE	P75	SE	P90	SE	P95	SE
								**Protein (g)**									
**Male**																	
20-30	76	145	34.2	94	11.6	104	10.4	121	8.33	143	7.51	167	10.4	191	15.8	206	19.9
31-50	84	121	41.4	65	8.2	74	7.6	91	6.49	115	6.3	144	9.8	176	16.4	197	21.8
50+	56	107	28.9	68	8.1	74	7.4	86	6.31	102	6.17	122	9.7	145	16.5	160	22.2
**Female**																	
20-30	111	80	13.3	59.9	5.3	63.9	4.6	71	3.63	79.5	3.21	88.8	4.3	97.8	6.4	103.6	7.9
31-50	91	77	19.7	46	5.9	52	5.2	63	4	76	3.47	89	4.7	102	6.9	111	8.6
50+	88	72	12.2	53.9	4.4	57.3	3.9	63.5	3.07	71.2	2.75	79.7	3.8	88.2	5.9	93.7	7.4
**All**	506	98	35.5	51	2.6	58	2.5	72	2.16	92	2.16	117	3.4	145	5.9	164	7.9
								**Lipid (g)**									
**Male**																	
20-30	76	94	17.4	68	7.6	73	6.7	82	5.3	93	4.7	105	6.4	117	9.5	125	11.9
31-50	84	90	28.1	50	6.4	57	5.8	69	4.9	86	4.5	106	6.8	127	11.0	141	14.3
50+	56	67	13.2	47.4	6.5	51.1	5.8	57.9	4.5	66.2	4.0	75.4	5.6	84.5	8.6	90.4	10.8
**Female**																	
20-30	111	65	8.6	51.3	4.4	54.1	3.8	59	2.9	64.7	2.5	70.6	3.2	76.1	4.5	79.5	5.5
31-50	91	61	16.0	38	4.5	42	4.1	50	3.3	60	3.0	71	4.3	82	6.6	90	8.4
50+	88	56	13.2	36.6	4.1	39.9	3.8	46.1	3.1	54.1	2.9	63.4	4.2	73	6.6	79.4	8.6
**All**	506	72	22.3	40	2.1	46	1.9	56	1.6	69	1.6	85	2.3	101	3.7	112	4.9

[1]Standard Deviation
[2]Standard Error

Table S3. Distribution of means and percentiles of usual micronutrients intake according to BMI and physical activity. Brazil, 2016-2017.

Parameters	*N*	Mean	SD[1]	P5	SE[2]	P10	SE	P25	SE	P50	SE	P75	SE	P90	SE	P95	SE
								**Calcium (mg)**									
**BMI (kg/m²)**																	
< 25	267	1290.4	379.0	751	47.9	844	43.9	1019	36.6	1247	34.3	1514	50.5	1793	80.8	1978	105.0
25 – 29.9	166	1382.0	359.8	864	62.9	955	57.2	1125	47.1	1344	43.6	1597	63.1	1858	100.0	2031	130.0
≥ 30	73	1249.6	445.9	606	102.0	718	91.6	929	73.6	1202	66.8	1520	101.0	1845	161.0	2057	206.0
**PHYSICAL**	**ACTIVITY**																
Yes	387	1350.9	414.0	761	41.3	862	37.8	1054	31.2	1304	29.1	1596	43.0	1900	69.1	2102	89.5
No	119	1202.3	319.8	737	73.4	820	66.2	974	54.1	1170	50.0	1396	71.8	1626	112.0	1777	144.0
								**Phosphorous (mg)**									
**BMI (kg/m²)**																	
< 25	267	1313.5	394.4	763	46.6	855	43.0	1031	36.2	1263	34.5	1541	51.7	1835	84.5	2035	111.0
25 – 29.9	166	1453.0	478.0	814	62.4	915	58.1	1111	50.0	1380	49.2	1714	77.6	2083	132.0	2340	178.0
≥ 30	73	1208.7	354.7	721	83.1	801	76.4	955	64.2	1161	61.4	1410	94.5	1678	158.0	1861	209.0
**PHYSICAL**	**ACTIVITY**																
Yes	387	1393.3	469.3	756	40.1	858	37.4	1057	31.9	1326	30.9	1656	48.0	2014	80.8	2260	108.0
No	119	1196.3	277.6	801	68.0	870	61.8	999	51.4	1165	48.4	1360	70.4	1563	112.0	1699	146.0
								**Magnesium (mg)**									
**BMI (kg/m²)**																	
< 25	267	497.9	123.5	315	16.7	348	15.0	410	12.0	487	10.7	574	15.1	661	23.5	717	29.9
25 – 29.9	166	523.2	146.8	315	21.9	351	19.9	418	16.4	506	15.3	609	22.9	718	37.2	790	48.5
≥ 30	73	438.2	120.6	260	30.3	292	27.1	352	21.4	427	18.9	512	27.5	598	43.3	653	55.3
**PHYSICAL**	**ACTIVITY**																
Yes	387	519.9	137.2	319	14.5	355	13.1	422	10.5	507	9.5	603	13.8	702	21.7	766	27.8
No	119	426.9	100.5	279	21.2	306	19.0	355	15.3	417	13.9	488	19.9	560	31.1	607	39.8

Table S3. Continued.

**Parameters**	***N***	**Mean**	SD[1]	**P5**	SE[2]	**P10**	SE	**P25**	SE	**P50**	SE	**P75**	SE	**P90**	SE	**P95**	SE
**Zinc (mg)**																	
**BMI (kg/m^2)**																	
< 25	267	11.9	4.2	6.4	0.5	7.3	0.4	8.9	0.4	11.2	0.4	14.2	0.6	17.6	1.0	19.9	1.4
25 – 29.9	166	13.6	5.9	6.7	0.6	7.6	0.6	9.5	0.5	12.4	0.6	16.4	1.0	21.2	1.9	24.8	2.6
≥ 30	73	11.4	3.8	6.5	0.8	7.3	0.8	8.7	0.7	10.8	0.7	13.4	1.1	16.4	2.0	18.6	2.7
**PHYSICAL**	**ACTIVITY**																
Yes	387	12.7	5.0	6.4	0.4	7.3	0.4	9.2	0.3	11.8	0.3	15.2	0.5	19.2	1.0	22.0	1.4
No	119	11.6	4.1	6.4	0.6	7.2	0.6	8.7	0.6	10.9	0.6	13.7	1.0	17.0	1.7	19.4	2.4
**Copper (mg)**																	
**BMI (kg/m^2)**																	
< 25	267	1319.8	546.8	760	47.7	843.0	43.0	995	35.5	1204	38.0	1497.0	69.1	1889	142.0	2231	239.0
25 – 29.9	166	1338.7	368.6	826	69.5	912.0	64.2	1076	54.5	1291	52.5	1550.0	78.0	1825	127.0	2013	167.0
≥ 30	73	1165.7	437.9	652	78.3	725.0	73.3	869	64.1	1075	66.9	1357.0	118.0	1711	234.0	1986	344.0
**PHYSICAL**	**ACTIVITY**																
Yes	387	1317.8	406.2	768	40.9	857.0	38.1	1028	32.9	1259	32.4	1542.0	49.6	1853	83.1	2068	111.0
No	119	1299.4	751.0	696	64.6	763.0	59.6	897	54.4	1105	67.8	1450.0	146.0	1990	351.0	2502	610.0
**Selenium (mcg)**																	
**BMI (kg/m^2)**																	
< 25	267	138.1	55.1	69	5.6	80.0	5.2	100	4.5	128	4.6	165.0	7.6	208	14.2	241	20.0
25 – 29.9	166	154.3	63.5	79	6.9	89.0	6.7	110	6.2	141	6.8	184.0	11.8	235	22.0	274	31.4
≥ 30	73	122.2	31.7	82	9.1	88.0	8.5	100	7.4	117	7.5	138.0	12.3	163	21.9	181	30.1
**PHYSICAL**	**ACTIVITY**																
Yes	387	149.5	62.5	76	4.9	87.0	4.5	107	3.8	136	4.1	177.0	7.4	228	14.1	267	20.3
No	119	116.8	35.8	70	6.5	77.0	6.1	91	5.4	111	5.5	136.0	8.8	164	15.2	184	20.7

Table S3. Continued.

Parameters	*N*	Mean	SD[1]	P5	SE[2]	P10	SE	P25	SE	P50	SE	P75	SE	P90	SE	P95	SE
								**Iron (mg)**									
**BMI (kg/m²)**																	
< 25	267	13.3	3.8	8.1	0.5	9.0	0.4	10.7	0.3	12.8	0.3	15.3	0.5	18.2	0.9	20.2	1.2
25 – 29.9	166	13.9	3.6	8.8	0.6	9.7	0.6	11.3	0.5	13.4	0.4	16.0	0.7	18.7	1.1	20.5	1.4
≥ 30	73	11.5	2.9	7.7	0.7	8.3	0.6	9.4	0.5	11.0	0.5	12.9	0.8	15.2	1.5	16.8	2.0
**PHYSICAL**	**ACTIVITY**																
Yes	387	13.4	3.8	8.2	0.4	9.1	0.4	10.8	0.3	12.9	0.3	15.5	0.4	18.4	0.7	20.3	0.9
No	119	12.6	3.5	8.0	0.6	8.7	0.6	10.1	0.5	12.1	0.5	14.5	0.8	17.3	1.4	19.2	1.9
								**Sodium (g)**									
**BMI (kg/m²)**																	
< 25	267	3.3	0.9	2.0	0.1	2.3	0.1	2.7	0.1	3.2	0.1	3.8	0.1	4.5	0.2	4.9	0.3
25 – 29.9	166	3.6	1.1	2.1	0.2	2.3	0.1	2.8	0.1	3.5	0.1	4.3	0.2	5.1	0.3	5.8	0.4
≥ 30	73	3.0	0.8	1.9	0.2	2.1	0.2	2.4	0.1	2.9	0.1	3.4	0.2	4.0	0.3	4.4	0.4
**PHYSICAL**	**ACTIVITY**																
Yes	387	3.4	1.1	2.0	0.1	2.2	0.1	2.7	0.1	3.3	0.1	4.1	0.1	4.9	0.2	5.4	0.3
No	119	3.1	0.9	2.0	0.2	2.2	0.2	2.5	0.1	3.0	0.1	3.6	0.2	4.3	0.3	4.7	0.4
								**Potassium (mg)**									
**BMI (kg/m²)**																	
< 25	267	2.8	0.8	1.6	0.1	1.8	0.1	2.2	0.1	2.7	0.1	3.3	0.1	3.8	0.2	4.2	0.2
25 – 29.9	166	3.0	0.9	1.8	0.1	2.0	0.1	2.4	0.1	2.9	0.1	3.5	0.1	4.1	0.3	4.6	0.4
≥ 30	73	2.6	0.7	1.6	0.2	1.7	0.2	2.1	0.1	2.5	0.1	3.0	0.2	3.6	0.3	3.9	0.4
**PHYSICAL**	**ACTIVITY**																
Yes	387	2.9	0.9	1.7	0.1	1.9	0.1	2.3	0.1	2.8	0.1	3.5	0.1	4.1	0.2	4.6	0.2
No	119	2.4	0.5	1.7	0.1	1.9	0.1	2.1	0.1	2.4	0.1	2.7	0.1	3.1	0.2	3.3	0.2

Table S3. Continued.

Parameters	*N*	Mean	SD[1]	P5	SE[2]	P10	SE	P25	SE	P50	SE	P75	SE	P90	SE	P95	SE
						**Manganese (mg)**											
**BMI (kg/m²)**																	
< 25	267	10.7	30.0	2.3	0.2	2.6	0.2	3.3	0.2	4.7	0.4	8.5	1.4	18.6	5.5	32.7	12.7
25 – 29.9	166	13.1	58.2	1.8	0.2	2.1	0.2	2.8	0.2	4.1	0.4	7.8	1.6	20.0	7.7	39.3	19.9
≥ 30	73	14.4	23.8	1.7	0.3	1.9	0.2	2.4	0.4	6.9	2.4	16.9	4.3	33.0	13.0	50.1	25.9
**PHYSICAL**	**ACTIVITY**																
Yes	387	11.3	14.0	2.2	0.1	2.5	0.1	3.1	0.1	5.1	0.6	14.7	2.6	28.6	3.2	37.0	4.9
No	119	10.8	51.8	1.6	0.2	1.8	0.2	2.3	0.2	3.2	0.3	6.1	1.4	15.6	7.3	31.4	19.3
								**Folate (mcg)**									
**BMI (kg/m²)**																	
< 25	267	352.8	116.6	192	13.3	219	12.3	269	10.6	337	10.3	419	15.5	510	25.6	567	33.8
25 – 29.9	166	361.5	91.7	232	17.9	254	16.4	296	13.7	351	13.1	415	19.2	483	30.9	528	40.2
≥ 30	73	317.5	71.8	218	23.4	234	21.3	266	17.8	308	17	358	25.7	412	42.2	449	55.7
**PHYSICAL**	**ACTIVITY**																
Yes	387	356.8	113.5	201	10.9	226	10.1	275	8.6	341	8.31	421	12.6	507	20.8	565	27.5
No	119	329.7	70.7	228	21.7	246	19.6	279	16.2	322	15.2	372	21.8	423	34.3	457	44.4
								**Vitamin A (mcg)**									
**BMI (kg/m²)**																	
< 25	267	1182.2	668.1	470	51.3	559	50.6	745	47.7	1025	53.5	1432	98.5	1974	204	2416	311
25 – 29.9	166	1288.0	610.3	560	83	667	79	875	68.9	1167	71	1559	122	2043	238	2420	348
≥ 30	73	1145.2	526.4	560	108	634	105	790	99.5	1025	111	1362	201	1793	399	2133	589
**PHYSICAL**	**ACTIVITY**																
Yes	387	1268.3	654.1	529	47.8	627	46.5	828	42.9	1124	47	1540	83.7	2072	166	2491	245
No	119	1032.9	495.7	460	77.3	538	74.8	695	69	927	75.2	1248	132	1651	255	1963	371

Table S3. Continued.

Parameters	*N*	Mean	SD[1]	P5	SE[2]	P10	SE	P25	SE	P50	SE	P75	SE	P90	SE	P95	SE
**Vitamin C (mg)**																	
**BMI (kg/m²)**																	
< 25	267	276.3	718.2	38	5.9	49	6.3	74	7.3	126	11.9	242	37.3	517	135	878	299
25 – 29.9	166	205.3	276.3	41	7.5	51	7.9	76	9.3	126	14.7	227	38.2	416	107	614	199
≥ 30	73	474.1	906.0	39	11.7	50	14.3	89	23.8	202	53.0	494	164	1084	490	1735	939
**PHYSICAL**	**ACTIVITY**																
Yes	387	201.7	252.9	43	5.2	54	5.7	82	6.5	132	9.1	226	21.9	396	62.4	576	116
No	119	546.0	2267.2	30	8.0	40	9.0	66	12.8	137	27.9	350	113	978	503	1899	1220
**Vitamin D (mcg)**																	
**BMI (kg/m²)**																	
< 25	267	4.6	3.0	1.4	0.2	1.8	0.2	2.7	0.2	3.9	0.2	5.8	0.4	8.2	0.9	10.1	1.4
25 – 29.9	166	5.0	2.4	1.9	0.3	2.3	0.3	3.3	0.3	5.9	0.4	6.3	0.5	8.2	0.8	9.5	1.1
≥ 30	73	4.4	1.9	1.9	0.5	2.3	0.5	3	0.4	4.1	0.4	5.4	0.7	6.9	1.2	7.9	1.6
**PHYSICAL**	**ACTIVITY**																
Yes	387	5.0	2.9	1.7	0.2	2.1	0.2	3	0.2	5.7	0.3	6.2	0.4	8.6	0.7	10.3	1.0
No	119	4.0	1.5	1.9	0.4	2.3	0.4	2.9	0.3	4.6	0.4	4.8	0.5	5.9	0.7	6.6	0.9
**Vitamin E (mg)**																	
**BMI (kg/m²)**																	
< 25	267	7.2	2.5	3.9	0.3	4.4	0.3	5.4	0.2	6.8	0.2	8.6	0.4	10.5	0.6	11.8	0.9
25 – 29.9	166	7.5	2.6	4.1	0.4	4.6	0.4	5.7	0.3	7	0.3	8.8	0.5	10.8	0.9	12.4	1.3
≥ 30	73	6.2	2.5	3.4	0.4	3.7	0.4	4.5	0.4	5.6	0.5	7.3	0.8	9.4	1.6	11	2.3
**PHYSICAL**	**ACTIVITY**																
Yes	387	7.5	2.8	4	0.2	4.5	0.2	5.5	0.2	7	0.2	8.8	0.3	11	0.6	12.7	0.9
No	119	6.1	1.9	3.6	0.4	4	0.4	4.7	0.3	5.8	0.3	7.1	0.5	8.6	0.9	9.7	1.1

Table S3. Continued.

Parameters	*N*	Mean	SD[1]	P5	SE[2]	P10	SE	P25	SE	P50	SE	P75	SE	P90	SE	P95	SE
								**Vitamin K (mcg)**									
**BMI (kg/m²)**																	
< 25	267	132.4	52.9	66	7.0	75	6.71	95	6.16	123	6.5	159	10.5	201	18.3	232	25.2
25 – 29.9	166	128.1	70.5	52	6.9	62	6.7	82	6.21	112	6.9	155	12.6	212	26.2	258	39.8
$\geq$ 30	73	84.9	24.1	52	9.0	57	8.35	68	7.09	82	6.8	99	10.3	117	17.1	129	22.6
**PHYSICAL**	**ACTIVITY**																
Yes	387	130.6	51.3	66	5.6	75	5.33	94	4.87	121	5.1	157	8.24	197	14.4	227	19.8
No	119	93.4	45.1	40	6.0	47	5.93	62	5.63	84	6.2	114	10.5	151	19.3	179	27.3
								**Vitamin B1 (mg)**									
**BMI (kg/m²)**																	
< 25	267	1.5	0.5	0.9	0.1	1.0	0.1	1.2	0.0	1.5	0.0	1.8	0.1	2.2	0.1	2.4	0.1
25 – 29.9	166	1.6	0.4	1.0	0.1	1.1	0.1	1.3	0.1	1.5	0.1	1.9	0.1	2.2	0.1	2.4	0.2
$\geq$ 30	73	1.4	0.4	0.9	0.1	1.0	0.1	1.1	0.1	1.3	0.1	1.6	0.1	1.9	0.2	2.1	0.2
**PHYSICAL**	**ACTIVITY**																
Yes	387	1.6	0.5	0.9	0.0	1.0	0.0	1.2	0.0	1.5	0.0	1.8	0.1	2.2	0.1	2.5	0.1
No	119	1.5	0.4	1.0	0.1	1.0	0.1	1.2	0.1	1.4	0.1	1.7	0.1	2.0	0.2	2.2	0.2
								**Vitamin B2 (mg)**									
**BMI (kg/m²)**																	
< 25	267	1.6	0.4	1.0	0.1	1.1	0.1	1.3	0.0	1.6	0.0	1.9	0.1	2.2	0.1	2.4	0.1
25 – 29.9	166	1.8	0.4	1.2	0.1	1.3	0.1	1.5	0.1	1.8	0.1	2.1	0.1	2.4	0.1	2.6	0.2
$\geq$ 30	73	1.5	0.4	0.9	0.1	1.0	0.1	1.2	0.1	1.5	0.1	1.8	0.1	2.1	0.2	2.4	0.3
**PHYSICAL**	**ACTIVITY**																
Yes	387	1.7	0.5	1.0	0.0	1.1	0.0	1.4	0.0	1.7	0.0	2.0	0.1	2.4	0.1	2.6	0.1
No	119	1.6	0.3	1.2	0.1	1.3	0.1	1.4	0.1	1.6	0.1	1.8	0.1	1.9	0.1	2.1	0.2

Table S3. Continued.

Parameters	*N*	Mean	SD[1]	P5	SE[2]	P10	SE	P25	SE	P50	SE	P75	SE	P90	SE	P95	SE
								**Vitamin B3 (mg)**									
**BMI (kg/m²)**																	
< 25	267	24.6	8.8	13.0	1.0	14.8	0.9	18.3	0.8	23.2	0.8	29.3	1.3	36.2	2.2	41.0	3.0
25 – 29.9	166	26.8	7.6	16.4	1.5	18.1	1.4	21.4	1.2	25.7	1.2	31.0	1.8	36.9	3.0	40.9	4.0
≥ 30	73	23.3	6.0	15.2	1.9	16.5	1.8	19.0	1.5	22.5	1.5	26.6	2.3	31.2	3.8	34.4	5.1
**PHYSICAL**	**ACTIVITY**																
Yes	387	25.8	9.5	13.7	0.8	15.5	0.8	19.1	0.7	24.2	0.7	30.8	1.2	38.2	2.0	43.5	2.8
No	119	23.0	6.0	14.6	1.6	16.0	1.5	18.7	1.3	22.2	1.2	26.4	1.8	30.9	2.9	33.9	3.8
								**Vitamin B5 (mg)**									
**BMI (kg/m²)**																	
< 25	267	5.4	1.4	3.5	0.2	3.8	0.2	4.4	0.2	5.3	0.1	6.3	0.2	7.3	0.3	8.1	0.5
25 – 29.9	166	5.7	1.5	3.6	0.2	4.0	0.2	4.7	0.2	5.5	0.2	6.6	0.3	7.8	0.5	8.6	0.6
≥ 30	73	5.0	1.3	3.3	0.3	3.6	0.3	4.1	0.2	4.8	0.2	5.7	0.4	6.7	0.7	7.5	1.0
**PHYSICAL**	**ACTIVITY**																
Yes	387	5.6	1.7	3.4	0.2	3.7	0.1	4.4	0.1	5.4	0.1	6.6	0.2	7.9	0.3	8.7	0.4
No	119	4.9	0.6	4.0	0.3	4.2	0.3	4.5	0.2	4.9	0.2	5.3	0.3	5.7	0.4	5.9	0.5
								**Vitamin B6 (mg)**									
**BMI (kg/m²)**																	
< 25	267	2.0	0.6	1.1	0.1	1.3	0.1	1.5	0.1	1.9	0.1	2.3	0.1	2.8	0.2	3.2	0.2
25 – 29.9	166	2.2	0.7	1.3	0.1	1.4	0.1	1.7	0.1	2.1	0.1	2.5	0.1	3.1	0.2	3.4	0.3
≥ 30	73	1.8	0.5	1.1	0.1	1.2	0.1	1.5	0.1	1.8	0.1	2.1	0.2	2.5	0.3	2.8	0.4
**PHYSICAL**	**ACTIVITY**																
Yes	387	2.1	0.7	1.2	0.1	1.3	0.1	1.6	0.1	2.0	0.1	2.5	0.1	3.0	0.1	3.4	0.2
No	119	1.8	0.5	1.1	0.1	1.2	0.1	1.4	0.1	1.7	0.1	2.1	0.1	2.5	0.2	2.8	0.3

Table S3. Continued.

Parameters	*N*	Mean	SD[1]	P5	SE[2]	P10	SE	P25	SE	P50	SE	P75	SE	P90	SE	P95	SE
						Vitamin B12 (mcg)											
**BMI (kg/m²)**																	
< 25	267	4.7	3.9	1.9	0.2	2.2	0.2	2.9	0.2	3.8	0.2	5.2	0.4	7.8	1.1	10.6	2.0
25 – 29.9	166	5.2	2.5	2.2	0.3	2.7	0.3	3.5	0.2	4.7	0.3	6.4	0.5	8.3	0.9	9.8	1.3
≥ 30	73	5.0	2.6	2.2	0.5	2.6	0.5	3.3	0.4	4.4	0.4	5.9	0.8	8.0	1.8	9.8	2.8
**PHYSICAL**	**ACTIVITY**																
Yes	387	4.7	2.5	1.9	0.2	2.3	0.1	3.0	0.1	4.1	0.2	5.7	0.3	7.7	0.5	9.3	0.8
No	119	5.8	4.4	2.2	0.3	2.5	0.3	3.1	0.4	4.4	0.5	6.8	1.2	10.5	2.8	13.8	4.4

[1]Standard Deviation

[2]Standard Error

Table S4. Distribution of means and percentiles of usual intake of energy and macronutrients according to BMI and physical activity. Brazil, 2016-2017.

Parameters	*N*	Mean	SD[1]	P5	SE[2]	P10	SE	P25	SE	P50	SE	P75	SE	P90	SE	P95	SE
								**Energy (kcal)**									
**BMI (kg/m²)**																	
< 25	267	1944	554.4	1154	66.8	1290	60.8	1548	50.1	1882	46.4	2272	68.7	2679	111.0	2950	144.0
25 – 29.9	166	2120	555.0	1333	89.4	1469	81.5	1724	67.5	2055	63.2	2445	93.6	2854	151.0	3128	198.0
≥ 30	73	1809	366.5	1274	121.0	1371	108.0	1549	87.2	1774	79.5	2031	114.0	2293	180.0	2466	232.0
**PHYSICAL**	**ACTIVITY**																
Yes	387	2012	568.7	1208	55.5	1345	50.6	1605	41.9	1944	39.2	2345	58.6	2765	95.2	3047	124.0
No	119	1895	471.8	1221	98.9	1339	90.2	1559	74.7	1843	69.7	2174	101.0	2519	162.0	2749	211.0
								**Carbohydrate (g)**									
**BMI (kg/m²)**																	
< 25	267	231	72.2	129	8.8	146	8.0	179	6.7	223	6.3	274	9.3	327	15.0	362	19.5
25 – 29.9	166	241	70.9	139	13.1	158	11.7	191	9.2	233	8.4	281	12.4	333	20.8	368	27.7
≥ 30	73	217	58.0	131	16.6	147	14.9	176	12.0	212	10.8	253	15.3	294	23.9	320	30.4
**PHYSICAL**	**ACTIVITY**																
Yes	387	234	71.8	131	7.4	149	6.8	183	5.6	226	5.2	277	7.6	330	12.1	364	15.7
No	119	226	66.0	132	14.8	150	13.2	181	10.3	219	9.3	264	13.9	312	23.6	345	31.6

Table S4. Continued.

Parameters	*N*	Mean	SD[1]	P5	SE[2]	P10	SE	P25	SE	P50	SE	P75	SE	P90	SE	P95	SE
								**Protein (g)**									
**BMI (kg/m²)**																	
< 25	267	95	33.7	49	3.6	56	3.4	71	2.9	90	2.9	113	4.4	139	7.4	157	9.9
25 – 29.9	166	107	36.3	58	5.0	66	4.7	81	4.1	101	4.1	127	6.4	155	10.9	174	14.6
≥ 30	73	89	27.8	54	5.8	59	5.4	69	4.6	83	4.7	102	7.9	124	14.7	141	20.9
**PHYSICAL**	**ACTIVITY**																
Yes	387	101	38.2	52	3.2	60	2.9	75	2.5	95	2.4	121	4.0	151	7.3	172	10.2
No	119	86	25.0	52	5.0	58	4.7	68	4.0	83	4.0	100	6.0	119	10.0	132	13.4
								**Lipid (g)**									
**BMI (kg/m²)**																	
< 25	267	70	20.7	40	2.9	45	2.6	55	2.2	68	2.1	82	3.0	97	4.7	107	6.0
25 – 29.9	166	78	24.6	44	3.9	50	3.6	60	3.1	75	3.0	92	4.4	111	7.3	123	9.6
≥ 30	73	62	6.9	51	6.3	53	5.4	57	4.1	62	3.4	66	4.6	71	7.0	74	8.7
**PHYSICAL**	**ACTIVITY**																
Yes	387	73	22.0	41	2.5	47	2.3	57	1.9	70	1.8	86	2.6	102	4.2	113	5.4
No	119	68	20.2	40	4.0	45	3.7	54	3.2	65	3.1	80	4.6	95	7.5	105	9.8

[1]Standard Deviation

[2]Standard Error

Table S5. Distribution of mean and percentiles of usual grams of food and beverages intake according to gender, age, BMI and physical activity. Brazil, 2016-2017.

Parameters	*N*	Mean	SD[1]	P5	SE[2]	P10	SE	P25	SE	P50	SE	P75	SE	P90	SE	P95	SE
**Male**																	
20-30	76	4075.5	822.89	2824	257	3064	226	3495	177	4020	155	4595	213	5159	322	5517	403
31-50	84	3754.4	811.65	2562	196	2779	174	3178	138	3679	124	4249	179	4828	284	5205	364
50+	56	3662.1	876.06	2358	264	2600	231	3039	179	3588	158	4207	230	4825	359	5220	453
**Female**																	
20-30	111	2987	642.5	1997	161	2190	141	2535	109	2950	94.2	3399	128	3831	190	4103	236
31-50	91	3148.7	738.91	2040	194	2247	172	2625	136	3091	121	3610	169	4125	258	4454	326
50+	88	2933.5	590.48	1987	166	2183	142	2525	105	2919	83.9	3326	111	3700	162	3928	198
**BMI (kg/m²)**																	
< 25	267	3296.8	867.95	2027	109	2255	98.5	2678	79.1	3213	71.1	3825	103	4446	163	4852	210
25 – 29.9	166	3556.3	816.77	2343	150	2567	133	2976	106	3487	95.1	4061	134	4635	208	5007	264
≥ 30	73	3244	768.21	2100	253	2349	208	2747	137	3180	111	3665	171	4211	313	4607	428
**PHYSICAL**	**ACTIVITY**																
Yes	387	3466.8	878.01	2168	94.6	2405	84.7	2842	67.5	3389	60.2	4007	86.1	4628	135	5031	172
No	119	3067.1	736.79	1998	150	2189	135	2542	110	2992	101	3510	146	4042	231	4392	297

[1]Standard Deviation

[2]Standard Error
